# A Japanese Boy With Spotted Fever and Overlapping Symptoms of Kawasaki Disease: A Case Report

**DOI:** 10.7759/cureus.51915

**Published:** 2024-01-08

**Authors:** Kosuke Sasaki, Kenji Yamada, Chihiro Matama, Daisuke Koike, Tomohiro Hirade, Junji Mashino, Fumihide Kato, Takeshi Taketani

**Affiliations:** 1 Department of Community Medicine, Shimane Prefectural Central Hospital, Himebara, JPN; 2 Department of Pediatrics, Shimane University Faculty of Medicine, Izumo, JPN; 3 Department of Pediatrics, Shimane Prefectural Central Hospital, Himebara, JPN; 4 Department of General Medicine, Shimane Prefectural Central Hospital, Himebara, JPN

**Keywords:** tosufloxacin, azithromycin, kawasaki disease, rickettsia japonica, japanese spotted fever

## Abstract

Japanese spotted fever (JSF) is a tick-transmitted infection caused by *Rickettsia japonica *(*R. japonica*), which is indigenous to Japan. Patients with JSF typically present with fever and spotted erythema on the palms and/or soles, and most of them have site(s) of tick bites. The prognosis is good, but some cases have a fatal course. Kawasaki disease (KD) is a systemic vasculitis with an unknown cause that is characterized by symptoms such as fever, conjunctival injection, oral findings, amorphous rash, rigid edema, and nonsuppurative cervical lymphadenopathy. Although the symptoms of JSF are partially similar to those of KD, case reports of JSF overlapping KD have never been internationally published. Herein, we report a boy with JSF and KD symptoms. A five-year-old boy presented with fever and rash after he had been on a mountain inhabited by *R. japonica*. On the fifth day, erythema was spotted mainly on his bilateral palms, bilateral cervical lymphadenopathy, rigid edema of his lower feet, and mild conjunctival injection appeared. Intravenous immunoglobulin (IVIG) therapy was performed because these symptoms satisfied five out of the six diagnostic criteria for KD. However, on the sixth day, the fever persisted, and then we readministered IVIG in addition to tosufloxacin and azithromycin since we found a tick-bite eschar, which suggested a complication of JSF. His symptoms resolved soon after this treatment. Coronary artery lesions were never observed. This case indicates that the *R. japonica* infection overlaps clinically with KD. Tosufloxacin and azithromycin should be considered to avoid the use of minocycline in younger patients with JSF.

## Introduction

Japanese spotted fever (JSF) is a tick-transmitted infection caused by *Rickettsia japonica* (*R. japonica*) [1]. Rickettsia is a genus of nonmotile, gram-negative, nonspore-forming, and highly pleomorphic bacteria, and *R. japonica* was first reported by Mahara et al. in 1984 [2]. Currently, more than 200 patients with *R. japonica* are identified annually in Japan (the number of patients in Japan is reported by the National Institute of Infectious Diseases [17]), and some fatal cases have also been reported. Patients with JSF typically present with high fever, headache, and characteristic erythema (especially spotted erythema on the palms and/or soles), and 90% of them have site(s) of tick bites [3]. The onset age varies, but infantile cases are relatively rare [4]. The diagnosis is made with paired serum testing using immunofluorescent assays and detection of *R. japonica* in tick bite eschars and clots using polymerase chain reaction (PCR). JSF is treated with antibiotics such as tetracyclines and/or new quinolones. When the treatment is inappropriate and/or delayed, disseminated intravascular coagulation (DIC) and death can occur; therefore, early diagnosis and appropriate treatment are important.

Kawasaki disease (KD) is a syndrome of unknown cause that was first reported by Tomisaku Kawasaki in 1967 and mainly affects children under five years of age [5]. KD causes systemic vasculitis, resulting in characteristic symptoms such as high fever, conjunctival injection, oral findings, including diffuse erythema of oral mucosa and strawberry tongue, amorphous rash, rigid edema or erythema of hands and feet (or peeling skin), and nonsuppurative cervical lymphadenopathy. The diagnosis is made when five or more of the six main symptoms are presented. Furthermore, in atypical (or incomplete) KD cases who have fewer than 5 of 6 symptoms, to look for other differential diagnoses abnormal laboratory findings, including not only elevated white blood cells (WBC) and C-reactive protein (CRP) but also sterile pyuria, elevated liver enzymes, and reduced serum albumin, should be checked. Coronary artery lesions (CAL), including coronary artery aneurysm formation, are serious complications. The recent incidence of KD in Japan was approximately 10,000 cases per year [18]. The initial treatment for KD consists of high-dose aspirin and intravenous immunoglobulin (IVIG). Early treatment after diagnosis of KD is important because coronary artery aneurysms occur in up to 20% of patients, and approximately 0.17% of patients die without treatment [6].

Because rickettsia infection and KD cases both present with febrile rash, JSF is an important differential diagnosis, but cases of KD associated with JSF have rarely been reported. Herein, we report a boy with JSF who presented with symptoms observed in KD, including fever, conjunctival injection, rash, hard edema, and cervical lymphadenopathy.

## Case presentation

**Figure 1:**
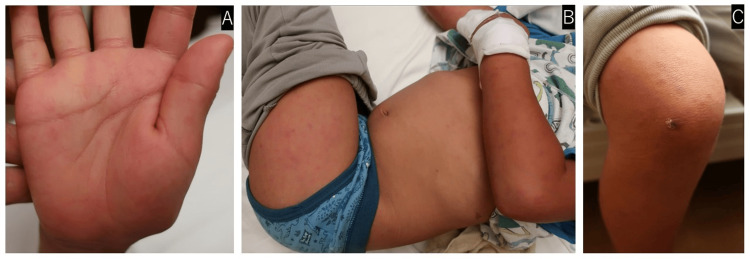
Skin findings on the limbs (A) Spotted erythema on the palms. (B) Spotted erythema on the extensor surfaces of the thighs and upper arms. (C) A tick-bite eschar on the right kneecap.

The patient was a five-year-old boy who did not have any pathological familial or past history. He did not have a pet, he did not take medicines, and he did not drink well water. Fever and rash were first observed on a certain day in July 2021, and amoxicillin was administered on the second day after fever in a pediatric clinic. Because he developed five of the main symptoms of KD, such as a fever of 39°C, spotted erythema all over the body, particularly on both palms (Figures 1A, 1B), bilateral cervical lymphadenopathy, rigid edema localized to his lower feet, and a mild conjunctival injection on the fifth day, he was initially diagnosed with KD and was referred to our hospital.

Upon admission to our hospital, his body temperature was 39.4°C, his pulse rate was 115/min, and his respiratory rate was 38/min. He did not present with oral mucosal changes or induration of a Bacille de Calmette et Guerin (BCG) scar. A complete blood count test showed a WBC count of 6,420 cells/μL, including 58% neutrophils; a red blood cell (RBC) count of 427 x104 cells/μL; a hemoglobin level of 12.1 g/dL; and a platelet count of 12.5 x104 cells/μL. His D-dimer level was 3.32 μg/mL (normal range, 0-0.99). Other abnormal laboratory findings included mild elevations of CRP (2.47 mg/dL, reference value <0.14), aspartate aminotransaminase (AST) (60 IU/L; normal range, 13-30), alanine aminotransferase (ALT) (36 IU/L; normal range, 10-42), and lactate dehydrogenase (LDH) (390 IU/L; normal range, 124-222) and mild decreases of total protein (5.9 g/dL; normal range, 6.6-8.1), albumin (3.0 g/dL; normal range, 4.1-5.1), and sodium (133.5 mmol/L; normal range, 138-145) (Table 1).

**Table 1:** Results of routine laboratory data on the admission * Reference values were used for adult patients in Shimane Prefectural Central Hospital. A bold type indicates an abnormal value. WBC: White blood cells, RBC: Red blood cells, PT: Prothrombin time, PT-INR: Prothrombin time-international normalized ratio, APTT: Activated partial thromboplastin time, FDP: Fibrin degradation products, AST: Aspartate aminotransferase, ALT: Alanine transaminase, LDH: Lactate dehydrogenase, CK: Creatine kinase, Na: Sodium, K: Potassium, CI: Cardiac index, CRP: C-reactive protein

		unit	reference*
Complete Blood Count			
WBC	6.42	x10^3^ /μL	(3.3-8.6)
Neutrophil	58%		(38.5-80.5)
Lymphocyte	36%		(16.5-49.5)
Atypcal lymph	0.5%		(<0.0)
RBC	427	x10^4^ /μL	(435-555)
Hemoglobin	12.1	g/dL	(13.7-16.8)
Platelet	12.5	x10^4^ /μL	(15.8-34.8)
Coagulation parameters			
PT	11.7	sec	
PT-INR	1.04		(0.85-1.15)
APTT	28.5	sec	(25-36)
D-dimer	3.32	μg/mL	(<0.99)
FDP	6.4	μg/mL	(<4.9)
Biochemical data			
Total protein	5.9	g/dL	(6.6-8.1)
Albumin	3.0	g/dL	(4.1-5.1)
Total bilrubin	0.3	mg/dL	(0.4-1.5)
AST	60	IU/L	(13-30)
ALT	36	IU/L	(10-42)
LDH	390	IU/L	(124-222)
CK	105	IU/L	(59-248)
Urea nitrogen	10.4	mg/dL	(8.0-20.0)
Creatinine	0.41	mg/dL	(0.65-1.07)
Na	133.5	mEq/L	(138-145)
K	3.8	mEq/L	(3.6-4.8)
Cl	99.1	mEq/L	(101-108)
CRP	2.47	mg/dl	(<0.14)

These findings indicated a high likelihood of resistance to IVIG, and Osaka, Kurume, and Gunma scores were negative [7-9]. Echocardiography showed no obvious findings of increased brightness, dilatation, aneurysm formation, or tortuosity in his coronary arteries. Virus antigen tests of SARS-CoV-2 (severe acute respiratory syndrome coronavirus 2) and adenovirus and serum antibody titers of cytomegalovirus and Epstein-Barr virus showed all were negative.

**Figure 2:**
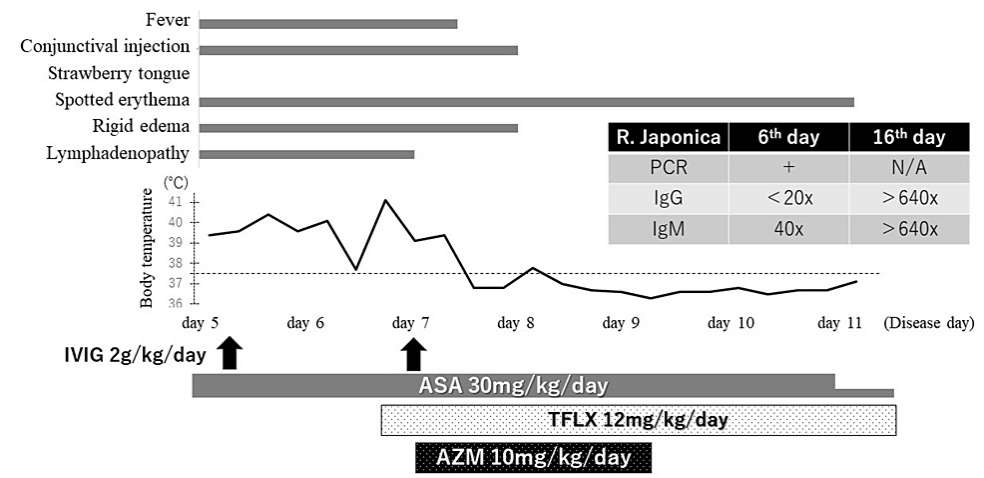
Clinical course after hospitalization IVIG: Intravenous immunoglobulin; ASA: Acetylsalicylic acid; TFLX: Tosufloxacin; AZM: Azithromycin.

Combination therapy consisting of IVIG (2 g/kg/day) and high-dose aspirin (30 mg/kg/day) was started based on the diagnosis of KD, but his symptoms did not improve 24 hours after the treatment. To reevaluate KD, his physical findings and medical history were rechecked. In fact, until two days before the onset of fever, he had spent two weeks near Mt. Kitayama, where infections of *R. japonica* had been observed. On the evening (sixth day of disease; second day of hospitalization), we found an approximately 5-millimeter tick-bite eschar on his right knee (Figure 1C). Although his parents noticed the eschar before his admission, they did not complain about it because they thought it was just an insect bite or a scrape. Tosufloxacin (TFLX) was also administered because JSF was suspected. Since the fever persisted despite the above treatments, IVIG was readministered as the second line of treatment for KD, and azithromycin (AZM) was added for JSF on the seventh day. After that, his symptoms, including fever, cervical lymphadenopathy, conjunctival injection, and rigid edema of his feet, were resolved. Low-dose aspirin (5 mg/kg/day) was started. After alleviation of the fever, AZM and TFLX were administered on third and fifth day, respectively. He was discharged on the eleventh day (Figure 2). A mild thrombocytosis (platelet, 39.8-40.2 x104 cells/μL) was exhibited from the 11th to the 16th day. Spotted erythema disappeared on the sixteenth day, and peeling skin was never observed. Low-dose aspirin was continued for three months, and no CAL was observed by his echocardiography during the observation period (approximately half year).

Subsequently, we were informed that *R. japonica* was detected using PCR in the eschar collected on the sixth day (we were unable to collect clots), resulting in the definitive diagnosis of JSF. The patient’s immunoglobulin G (IgG) and immunoglobulin M (IgM) titers of *R. japonica* were <20x and 40x on the sixth day, respectively, and they were both >640x on the sixteenth day.

## Discussion

We present a case of JSF associated with KD symptoms, which has only been reported in Japanese domestic journals and has not previously been reported in international journals. Because the main symptoms of KD, such as high fever, cervical lymphadenopathy, conjunctival injection, rigid edema of the lower feet, and spotted erythema mainly in the palms, were observed in this case, KD was initially diagnosed, but IVIG and aspirin were not effective. Subsequently, JSF was definitively diagnosed, and the patient was cured by treatment with TFLX and AZM. This case indicates that the infection caused by *R. japonica* can result in overlapping symptoms with KD.

Interestingly, only four pediatric cases with JSF associated with KD-like symptoms have ever been reported in Japanese domestic journals (Table 2) [10-12].

**Table 2:** Cases of Japanese spotted fever presenting similarly to Kawasaki disease reported in Japanese domestic journals Y: Year(s); m: Month(s); M: Male; F: Female; IVIG: Intravenous immunoglobulin; AZM: Azithromycin; TFLX: Tosufloxacin; MINO: Minocycline; CTX: Ceftriaxone; admin: Administration. Oral manifestation includes strawberry tongue, or erythema of oral mucosa. Extremity changes indicate rigid edema and erythema on the palms and soles or tips of the fingers and toes. Regarding the treatment of aspirin, the information was not described in Cases 1 and 4, and aspirin was used in Case 2, while it was not in Case 3.

Case	Age	Sex	Six main symptoms of Kawasaki disease						Spotted erythema on palms and soles	Initial treatment → 2nd-line therapy	Response to treatment	History of contact with endemic area	Reference
			Fever	Erythema	conjunctival injection	Oral manifestation	Cervical lymph-adenopathy	Extremity changes					
1	2m	F	+	+	-	-	-	-	+	IVIG + ABPC + CTX → AZM + MINO	Cured after 10 days of 2nd-line therapy	-	Nashida, et al. [7]
2	1y2m	M	+	+	+	+	+	-	+	IVIG + AZM	Fever resolved the day after initial therapy	-	Nashida, et al. [7]
3	4y	F	+	+	-	+	+	-	+	MINO	Fever resolved the day after initial therapy	+	Miyazono, et al. [8]
4	9y	F	+	+	+	+	+	+	+	IVIG + CTX → MINO	Fever resolved 3 days after 2nd-line therapy	+	Okuno, et al. [9]
Our case	5y	M	+	+	+	-	+	+	+	IVIG twice → AZM + TFLX	Fever resolved 3 days after 2nd-line therapy	+	

Although all four patients presented with fever and rash, other symptoms varied from case to case. A reddened BCG scar has not been described or observed in any cases. Rigid edema and a strawberry tongue that were atypical for JSF were observed, suggesting that JSF and KD cannot be distinguished by only clinical symptoms. This may be because some of the main symptoms of KD are subjectively estimated. In fact, our patient initially had an odd presentation for KD in retrospect because his conjunctival injection and rigid edema were mild and atypical for KD. In our case, the workup to diagnose JSF included finding a tick-bite eschar and obtaining a history of travel, particularly determining whether the patient had gone to areas where ticks are endemic. Furthermore, spotted erythema on the palms (Figure 1A) is typical for JSF but atypical for KD. Other characteristic features of JSF erythema include an onset immediately after fever, spreading from the limbs to the trunk, a lack of itchiness and pain, and transformation into petechia within a few days [1]. These presentations, including tick-bite eschar and the history of migration to areas where ticks are endemic, might be momentous to distinguish between JSF and KD. The most important data for the identification of JSF are serological assessments and PCR tests. Additionally, Tsutsugamushi disease is also a differential diagnosis of JSF. Mahara et al. have already reported the differences between JSF and Tsutsugamushi disease as follows: In JSF, as compared to scrub typhus, there are 1) spotted erythema on the palms, 2) a predilection for warmer seasons, 3) eschars smaller than 1 cm, and 4) rare lymphadenopathy [1]. In other words, spotted erythema on palms can be considered a crucial distinguishing point between JSF and KD, or Tsutsugamushi disease.

In our case, treatment with IVIG was considered reasonable and proper, even though the patient appeared to have a presentation that was odd for KD, as mentioned above. This case also exhibited hypoalbuminemia and hyponatremia, which were helpful findings to suspect KD in Japanese diagnostic guidelines, indicating a few contradictions as KD. By contrast, thrombocytopenia, a normal WBC count, and a mild increase in CRP were findings more consistent with JSF than KD in retrospect. Nevertheless, the date of admission was the fifth day of onset, and delaying treatment could have led to complications of CAL. Because inflammation of the intima and adventitia of the coronary arteries occurs on the 6th-8th days of the disease, complete treatment is generally required until the 10th day to prevent CAL [13]. In fact, IVIG was used in three of four cases of JSF associated with KD (Table 2). The patient in the other case (patient 4) was initially diagnosed with JSF and was treated with only minocycline (MINO) because she had a familial history of siblings with JSF. Once KD is diagnosed, treatment with IVIG cannot be avoided, even if JSF is also diagnosed. This is because CAL has been reported to be associated with infections of Rickettsia [14].

It is difficult to select antibiotics for younger patients with JSF because some antibiotics have side effects specific to children, and long-term follow-up is required to detect the side effects. MINO is first used for adult patients with JSF, and a new quinolone is concurrently added in severe cases [3]. However, these antibiotics are not generally recommended for younger (less than eight years old) patients because they have negative effects on their teeth, joints, and/or central nervous system. Although doxycycline can be empirically used for children, the toxicity of tooth bud formation is possible. In our case, TFLX and AZM were administered to avoid these adverse effects. Although the effect of TFLX on rickettsia infection has barely been reported [15], TFLX is a safe new quinolone, even for children. AZM has been reported to be as efficacious as doxycycline for Mediterranean spotted fever and emerging tick-borne rickettsiosis [16]. Furthermore, a case report showed the efficacy of AZM for JSF in a Japanese domestic journal (Tsuyoshi Higuchi et al., Shounika Rinsyou, 2009). Although the evidence of TFLX and AZM for JSF is limited, these drugs might be useful in younger patients with JSF.

## Conclusions

This case demonstrates that the *R. japonica* infection can overlap with KD. The most differential point between JSF and KD is spotted erythema on palms and soles. Thrombocytopenia is typical for JSF but atypical for KD. AZM and TFLX should be considered to avoid treatment with tetracycline antibiotics in younger patients with JSF.
